# Limiting global warming to 1.5 to 2.0°C—A unique and necessary role for health professionals

**DOI:** 10.1371/journal.pmed.1002804

**Published:** 2019-05-14

**Authors:** Edward W. Maibach, Mona Sarfaty, Mark Mitchell, Rob Gould

**Affiliations:** 1 Center for Climate Change Communication, George Mason University, Fairfax, Virginia, United States of America; 2 Medical Society Consortium for Climate and Health, Fairfax, Virginia, United States of America

## Abstract

In an Editorial, Edward Maibach and colleagues discuss the important role of health professionals in future responses to threats of climate change.

In the 2015 Paris Climate Agreement, 195 nations agreed to work together to hold the rise in global temperature to “well below 2 degrees Celsius.” Recent research by the Intergovernmental Panel on Climate Change (IPCC) suggested the need for an even more ambitious goal: limiting the warming to 1.5°C to avert the considerable risks to human health, livelihoods, food and water supplies, security, and economic growth that are likely with 2°C of warming [1]. Unfortunately, the emission reduction commitments made thus far under the Paris Climate Agreement are grossly insufficient to hold global warming to 2°C, much less to 1.5°C [2].

Therefore, this moment is one of extraordinary consequence. Actions taken by all nations over the next decade will determine whether global health will continue to improve or whether it will instead decline—possibly catastrophically so—as a result of climate change.

## Background

Numerous recent scientific assessments—including the IPCC 1.5°C report [1] and the *Lancet* Countdown [3]—have made clear that health harms of climate change are no longer just a future threat: they are our current reality. These harms include illness, injuries, and deaths from increasingly dangerous weather (including extreme heat, precipitation, and flooding), worsening air pollution, the spread of infectious diseases, increases in food- and water-borne illnesses, reduced nutrition, and profound mental health harms caused or made worse by traumatic climate events.

Emissions of heat-trapping carbon pollution reached an all-time high in 2018 [4]. Global warming and related climate changes are occurring at an accelerating pace [5], as are the health harms they cause [3]. Of greatest concern—if one or more possible tipping points in the world’s climate systems are triggered—these accelerating harms may occur in a nonlinear manner, with high potential for catastrophic consequences to health and prosperity [6].

This prospect should be of grave concern to—and should serve as a clarion call for—all health professionals. As health professionals, we pledge to do no harm. We often aspire further, to serve humanity by preventing needless harm and suffering and to foster conditions that give every person a fair chance at the blessings of good health—especially those who are most vulnerable. Climate change directly imperils these aims.

Our collective contributions to human health and well-being over the past century have been remarkable. We have ushered in major improvements in health and longevity. Now, an entirely new challenge demands our full attention—the challenge of stopping and protecting people against the harms of climate change. Over the next decade, the nations of the world must embrace and accomplish three difficult but achievable objectives so that human civilization and the ecosystems on which we depend can continue to flourish: creating a clean energy economy, drawing down excess atmospheric carbon, and preparing for and adapting to health impacts.

## Three global objectives: Clean energy, carbon drawdown, and preparedness

The world’s transition to a clean energy economy must be greatly accelerated (i.e., nearly 100% clean energy for everything—heating, cooling, transportation, manufacturing, and agriculture). This can be done with wind, solar, geothermal, hydro, improved energy storage capabilities, smart electrical grids, and nuclear energy—although there are legitimate concerns about the inclusion of nuclear energy. This global transition has already started but must be greatly accelerated and must be completed within the next the next few decades. The Solutions Project has developed potentially viable plans for every state in the United States and for most nations in the world to demonstrate the feasibility of this objective [7,8].

The excess heat-trapping pollution in our skies must be harvested and put back into the ground and/or used to make solid products—reducing this pollution to the levels seen in preindustrial times, a reduction of about one-third from current levels. This can be done through natural and technological carbon-capture technologies, including and perhaps especially improved land use, forestry, and agricultural practices. This is a global project that will take decades to accomplish, but the sooner it begins at scale, the sooner levels of heat-trapping pollution will begin to drop. Project Drawdown has identified a range of the most cost-effective approaches that are ready for widespread adoption [9].

Every community, state, and nation must prepare itself for the unavoidable health impacts of climate change that are already happening and will continue to occur with greater frequency and severity over the next several decades (or longer)—as a result of inertia in the climate system. In the US, for example, the Centers for Disease Control and Prevention’s (CDC) Building Resilience Against Climate Effects (BRACE) framework provides recommended practices that can guide communities in this effort [10]. The health impacts of climate change—and therefore the most important interventions—vary from region to region, and the evidence about the effectiveness of many intervention options is relatively thin, but evidence-based interventions that are relevant to many communities include mosquito abatement programs (e.g., source reduction, larvicide, adulticide), hydration and access to cooling during extreme heat events, general remediation of flood damage to homes and other buildings, and indoor air filtration downwind of wildfires [11].

## Health professionals can make a critical difference

Health professionals and the organizations that represent us must become tireless champions for these objectives and for the global agreements that will enable them—especially the Paris Climate Agreement and the Sustainable Development Goals. In a world rife with rapidly eroding trust, we remain trusted honest brokers who place public interests above all others [12]. In this role, we must educate and advocate with people in our communities, civic leaders, and policy makers in government and industry—in our city or county, our state, our nation, and on the global stage—until the objectives have been embraced and achieved. The only way to achieve these objectives is through policy changes that will prioritize human health and sustainable ecosystems and will shift the burden of pollution and other forms of ecosystem degradation back onto those who are responsible, drawing on the common-sense principle of “polluter pays.” The Australian National Climate and Health Framework provides a compelling example of what the health community can and must do [13], as does the recent Call to Action on Climate and Health by Canada’s health professionals [14].

The voices of health professionals as champions and advocates for human health and sustainable ecosystems—in communities, states, nations, and global negotiations—is imperative. When our voices are absent, those most loudly heard by the public and policy makers are those of major corporations—especially fossil fuel companies whose business models are currently tied to the extraction and pollution that is directly harming human health and is jeopardizing the very possibility of continued human prosperity. The claim that, for the foreseeable future, the fossil fuel economy is the only viable economy is made in full knowledge that fossil fuel use causes irreparable harm to human health and to the earth’s climate system, on which all human life and prosperity depends.

Those with strong vested interests in the fossil fuel economy are likely to assert that we health professionals should “stick to our own lane” and stay out of complicated policy dialogues that we are ill-prepared to understand, much less advance. For us to acquiesce to that assertion would be irresponsible: protecting the health and well-being of people individually and collectively is our lane. We need not become experts in areas of policy outside our lane. Rather, we must only participate in the relevant policy discussions to ensure that the existentially important objectives we are advocating for are properly and fully considered. Failure to protect the health of people in this generation—and potentially the next hundred generations—is not an option.

In addition, we should lead by example—in our practices, our hospitals, our health departments, and our own lives. Organizations have already arisen from within our ranks—including Health Care Without Harm, Practice Green Health, and My Green Doctor—that are helping countless numbers of our peers to lead by example. We should all embrace this opportunity—both because the practice of healthcare and public health should not continue contributing to the problem and because leading by example is the most effective form of leadership.

## Conclusions

An ancient proverb holds that wrapped in every crisis is an opportunity. The crisis of climate change is a perfect example. Those who argue that responding to the climate challenge is risking our prosperity have it exactly wrong. That is because the actions that must be taken will not only ensure the long-term viability of human civilization but will also soon produce greater health and wealth for all nations that rise to the challenge. Our air and water will be cleaner, our health better, our productivity greater, and our economies stronger. Climate solutions are health solutions, and health solutions are economic solutions. Health professionals are among the people best positioned to make sure that the public and policy makers understand this. Carpe diem.

## Abbreviations

BRACE: Building Resilience Against Climate Effects
CDC: Centers for Disease Control and Prevention
IPCC: Intergovernmental Panel on Climate Change
